# Tumor Necrosis Factor Alpha Induces a Serotonin Dependent Early Increase in Ciliary Beat Frequency and Epithelial Transport Velocity in Murine Tracheae

**DOI:** 10.1371/journal.pone.0091705

**Published:** 2014-03-13

**Authors:** Sebastian Weiterer, Dagmar Schulte, Sabrina Müller, Thomas Kohlen, Florian Uhle, Markus A. Weigand, Michael Henrich

**Affiliations:** Department of Anaesthesiology, Intensive Care Medicine, Pain Therapy, Justus-Liebig-University Giessen, Giessen, Germany; Research Center Borstel, Germany

## Abstract

The tracheal epithelium prevents via its highly effective clearance mechanism the contamination of the lower airways by pathogens. This mechanism is driven by ciliary bearing cells which are not only in contact with the gas phase; in addition they are also influenced by inflammatory mediators. These mediators can alter the protective function of the epithelium. Since the pro-inflammatoric cytokine tumor necrosis factor-α (TNF-α) plays a pivotal role within the inflammatory cascade, we investigated its effect onto the tracheal epithelium measured by its ciliary beat frequency and the particle transport velocity. In organ explant experiments the ciliary beat frequency and the particle transport velocity were measured under the application of TNF-α using tracheae from male C57BL6J mice. We observed a dose dependent TNF-α induced increase of both particle transport velocity and ciliary beat frequency. Knock out mice experiments made evident that the increase was depended on the expression of tumor necrosis factor receptor 1 (TNF-R1). The increases in ciliary beat frequency as well as the accelerated particle transport velocity were either inhibited by the unspecific serotonin antagonist methysergide or by cyproheptadine a specific 5-HT_2_ receptor antagonist. Thus, acetylcholine antagonists or nitric oxide synthase (NOS) inhibitors failed to inhibit the TNF-α induced activation. In conclusion, TNF-α may play a pivotal role in the protection of lower airways by inducing ciliary activity and increase in particle transport velocity via TNF-R1 and 5-HT_2_ receptor.

## Introduction

The mucociliary clearance is an important defence mechanism of the lower respiratory tract. This innate protector clears the airway surface area from debris, infectious particles and pathogens and is propelled by directed function of ciliary bearing cells integrated in the airway epithelium. In addition to the mechanical defence mechanism, a specific composition of mucus with a balanced mixture of salt and water is an important regulation factor for successful airway clearance. A defect of the cystic fibrosis transmembrane conductance regulator (CFTR) gene causes a dysfunction of the CFTR-protein. This protein defect, which regulates the salt water balance, leads to dehydration of the epithelia lining airway surface and results in an ineffective clearance, formation of local inflammation and an increase of chronic airway infections [1] [2]. These mechanisms show the relevance of the epithelial cilia function and the clearing system in connection with the innate immune system.

It is generally accepted that the protective beating function of ciliary bearing cells is modulated by different stimuli. In addition to cyclic adenosine monophosphate (cAMP) and intracellular calcium concentration, cyclic guanosine monophosphate (cGMP) seem to be important messengers to activate phosporylation and mediate an increase in cilliary beat frequency [3]. Cholinergic modulators, vasoactive peptides or nitric oxide (NO) all seem to induce the synthesis of cGMP via activation of soluble guanylate cyclase [4].

Among epithelial cells it has been assumed that other cell types e.g. activated mast cells and platelets affect the mucociliary transport via the endogenous mediator serotonin that can act as an activator of ciliary beat function [5] [6]. It seems obvious that ciliary function is constantly controlled by physiological neuromediators like acetylcholine or noradrenalin released from autonomic nerve fibres or paracrine from other cell types [7] [8].

Beside this constant regulation of cilia bearing cells, further modulators can alter ciliary function depending on physiological or patho-physiological requirements. Among patho-physiological processes inflammation leads to activation of the immune system and early in this mechanism cytokines are generated and released, which then can cause imbalances of organ functions. These cytokines released during inflammation or infection may also influence mucociliary clearance capacity [9].

Within in the family of pro-inflammatory cytokines, TNF-α plays a key role in activation of immune cells integrated in the cascade of host response to infections. However, TNF-α has not only an impact on genuine immune cells, it also initiates functions of cells associated to the immune system that provide specific functions in the pro-inflammatory response. Beside cells of the innate and adaptive immune system, many other non-immune cells are also capable of producing TNF-α like e.g. endothelial cells, neuronal cell and cardiac myocytes. The effects of TNF-α are mediated mainly via TNF-R1 which is found on many cell types and TNF-α receptor-2 (TNF-R2) that is exclusively expressed by immune cells. Upon binding, TNF-α upregulates pro-inflammatory gene expression via different signal transduction cascades including NF-κβ or MAP kinase [10].

Nevertheless, the entire effects of cytokines onto the clearing system of lower airways are still poorly understood. The tracheal epithelium may be attributed to the immune system first by its passive barrier function to block microbial intrusion, and second by its active clearing mechanism driven by ciliary cells. For this reason, we analysed the short term effects of TNF-α onto the functions of tracheal epithelial cells by recording ciliary beat frequency (CBF) and epithelial particle transport velocity (PTV) in murine tracheae.

## Materials and Methods

### Preparation of tracheal segments and imaging

In the present study we used male C57BL6J (Charles River) mice aged between 12 and 15 weeks (25–35 g). In a further series of experiments we employed C57BL6J TNF-R1 knock out mice (Jackson Laboratory, B6.129-Tnfrsf1a^tm1Mak^/J), which are established animals for mice deficient in TNF-R1 with a C57BL6J background strain [11] [12]. For all experiments care and use of the animals were performed according to the German guidelines. The protocol was approved by the Animal Welfare Office of the Justus-Liebig-University Giessen (Permit Numbers: 428_AZ 306 and 443_M 932). The animals were sacrificed by inhalation of an overdose isoflurane (Baxter, Unterschleiβheim, Germany) in a closed chamber. Immediately after confirmation of death, the trachea was removed by opening the thorax via a parasternal incision and median incision of the throat. In situ, the trachea was carefully explanted by cutting cranial to the bifurcation and caudal of the larynx. The trachea was then directly transferred into a Delta T culture dish (Bioptechs, Butler, PA, USA) filled with 2 ml of cooled (4–8°C) HEPES-buffer (pH 7.4). To enable proper positioning of the trachea inside the dish, the bottom was previously coated with Sylgard polymer (Dow Corning, Wiesbaden, Germany). Organs were fixed using two fine minutiae (Fiebig Lehrmittel, Berlin, Germany) in a position in which the cartilage arches were facing the Sylgard while the musculus trachealis was facing upwards. This allowed the fine preparation including the removal of connective tissue and surrounding blood vessels by employing Vannas-Tübingen spring scissors (FST, Heidelberg, Germany). Finally, the pars musculus membranacea trachealis was cut in longitudinal direction allowing a direct visualisation of the respiratory epithelium while the tissue remained submerged in the buffer solution. The HEPES buffer was replaced by 2 ml of fresh HEPES buffer pH 7.4, 30°C and the dish was transferred to the stage holder of an upright transmission light microscope (BX50 WI, Olympus, Hamburg, Germany) within 30 min after euthanasia. The microscope was equipped with a temperature control unit maintaining a constant temperature at 30°C throughout each experiment in the centre of the dish. The imaging was performed using a Till Vision imaging software (Till Photonics, Gräfeling, Germany).

### Measurement of particle transport velocity

**Figure 1:**
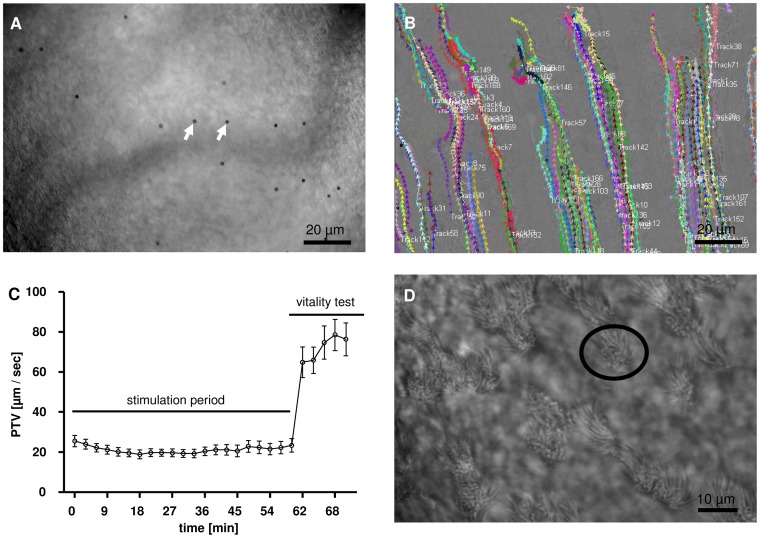
**A, B:** Microscopy for PTV measurements. Section of microscopic top view on the tracheal epithelium between 2 cartilages for PTV measurement (20x lens). **A:** Arrows indicate the applied particles (Dynabeads) **B:** Recorded particle tracks for PTV calculation. **C:** Representative experimental setup: The stimulation phase started 60 minutes after organ explantation. Measurements were performed over a period of 60 minutes (stimulation period). Vitality of the ciliary cells was tested by ATP application (10^−5^ M) over 10 minutes (vitality test) (Control group, n = 5, Mean ± S.E.M). **D:** Microscopy for CBF measurements**.** Section of microscopic top view between 2 cartilages for CBF measurement (40x lens)**.** Encircled area shows cilia bearing epithelial cell.

The particle tracking experiments were performed as described before [5]. For measurement of PTV, 4 μl Dynabeads (mean diameter between 2.8 μm to 4.5 μm; Dynal Biotech GmbH, Hamburg, Germany) were added prior to each measurement into the bath solution. Following, the surface of the tracheal epithelium was focused via a 20 x water immersion lens (Olympus) in bright field mode. To identify the transported Dynabeads, an area between two cartilages was selected as shown in figure 1A. This allowed recordings avoiding significant contrasts of the image brightness. At each time point short movie sequences with high sampling rates were recorded and subsequently analysed using *Image Pro Plus* analysis software. At the end of each individual experiment a viability proof of the ciliated cells in the trachea epithelium was performed by the application of ATP (100 μM) leading to maximal ATP-induced CBF increase. In each experiment tracks of approximately 200 to 400 particles were evaluated at certain pre-defined time points (figure 1B). At these time points 200 images were acquired during a period of 16.726 s. a background subtraction was performed pixel-by-pixel and non-moving objects were excluded (200 images/16.726 s  =  1 image/ 83.63 ms). Via this subtraction the formerly images of the darker polysterene Dynabeads were converted into bright images. Conversion into a binary image with a threshold that allowed to distinguish between the bright bead images and the dark background. Reduction of the 12 bit film to an 8 bit film on a greyscale enabled to follow individual particles via the TiLLvisTRAC software (Till Photonics) and calculating an average PTV (figure 1C).

### Measurement of ciliary beat frequency

To measure the CBF the epithelial cell layer of tracheae was focused using a 40 x objective (Zeiss, Jena, Germany) (figure 1D). Short movie sequences (9.523 s) were recorded with an EHD SMX-150 M CMOS camera (EHD Imaging, Damme, Germany). For evaluation of CBF 10 cells from each individual trachea (n  =  6) were chosen and light-dark-transmissions were analysed with Fourier-transformation analysis using 1000 images per time point (105 images/s). The analysis was achieved via fast Fourier transformation by administration of Autosignal 1.7 (SeaSolve Software Inc., San Jose, CA, USA).

Viability of the tissue was ensured by adding ATP at the end of each experiment. If the response to ATP failed the experiment was not included into further statistical analysis.

### Drugs and buffer solutions

The following drugs were administered: ATP (100 μM), methysergide (100 μM), WAY-100635 (1 μM), L-NAME (100 μM), L-NMMA (100 μM), atropine (1 μM) all from Sigma (Deisenhofen, Germany) cyproheptadine (5 μg/ml) (TOCRIS Bioscience, Bristol, UK).

Application of drugs taken from a stock solution (see above) was performed with a pipette into the organ bath solution; end concentration was achieved by gently mixing the buffer with a pipette. All preparation and experiments were carried out in HEPES solution consisting of : 20 mM HEPES, 4,5 mM KCl, 2,5 mM CaCl_2_, 11 mM Glucose, 140 mM NaCl, 1 mM MgCl_2_, pH was adjusted to 7.4 at 30°C or 4–8°C for tissue preparation using NaOH (4 molar).

TNF-α (PeproTech, Rocky Hill, New Jersey) was taken out of a stock solution (100 ng/μl, diluted in water) that was freshly prepared for each experimental day.

### RT-PCR

For PCR analysis mRNA was isolated from tracheae that had either been stimulated with TNF-α during PTV/CBF experiments or untreated organs as control. For further positive controls we isolated peripheral blood mononuclear cells (PBMC).

The tissue was processed using a bead mill homogenizer (Cryo Mill NM301, Retsch, Haan, Germany). Afterwards the supernatant was transferred into a gDNA eliminator column and then centrifuged with 8000 g for 30 s. Immediately followed by the mixture with 600 μl ethanol 70% and then 700 μl of this mixture was placed in a RNeasy Plus Mini Kit (Qiagen, Hilden, Germany) column. Two further centrifuge steps followed each at 8000 g for 15 s and in between the solution was resuspended with 700 μl RW 1 buffer solution. Thereafter 500 μl RPE buffer was added and the suspension centrifuged by 8000 g for 15 s, followed by a 2 min centrifugation. Eventually the solution was transferred into a 1.5 ml vessel and diluted with 50 μl RNase free water followed by a final centrifugation step at 8000 g for 15 s.

Transcription of RNA to cDNA was conducted using the Quantitect RTranscription kit (Qiagen, Hilden, Germany) by transferring 1 μg RNA into 2 μl gDNA Wipeout Buffer and dilution with RNase free water up to a total volume of 14 μl followed by incubation at 42°C for 2 min and further addition of 1 μl reverse transcriptase, 4 μl Quantiscript RT buffer and 1 μl RT primer omitted in control experiments. The transcription was then performed at 42°C for 20 min and stopped by heating up to 92°C for 3 min. The obtained cDNA was stored at –80°C until further PCR experiments.

PCR experiments were performed using the Promega-PCR-kit, the master mix consisted of 50 μl flexi buffer, 20 μl MgCl_2_, 5 μl dTNPs, 5 μl upstream primer and 5 μl downstream primer 1.25 μl DNA polymerase and 158.75 μl nuclease free water.

The primers used were as follows: TNF-R1 s: ATCTGCTGCACCAAGTGCC, as: TGCATGGCAGTTACACACG; TNF-R2 s: TACCAAGGGTGGCATCTCTC, as: TCCTGGGATTTCTCATCAGG. Additional 5 μl of template cDNA was added to the reaction solution, controls were conducted by the application of 5 μl nuclease-free water. The PCR was performed in a Mastercycler gradient (Eppendorf, Hamburg, Germany) for 40 min. administering the following cycle protocol: 95°C for 2 min followed by 95°C for 1 min then 56°C for 1 min afterwards 72° for 1 min and for 30 cycles, followed once by 72°C for 5 min and eventually cooling to 4°C. PCR products were evaluated by gel electrophoresis using 2% agarose gel, each trace was loaded with 25 μl of the PCR product and then started with 95 V for 20 min (Peqlab Biotechnology GmbH, Erlangen, Germany). The evaluation was achieved in a chemiluminescence imager (Fusion – SL 3500 WL) employing the Fusion software (both Vilber Lourmat, Eberhardzell, Germany).

Positive controls were performed by using RNA that was harvested from mice PBMC by small canules out of the heart after the animals had been sacrificed with isoflurane. The blood sample was transferred into a small glass tube containing 3 ml Ficoll and then centrifuged at 1500 g at 10°C for 30 min. Afterwards the cell layer containing the PBMC was pipetted into a fresh glas tube that was filled with phosphate buffered saline (PBS) to 10 ml and then centrifuged at 10°C at 6000 g for 10 min. The supernatant was discarded and the cell pellet was resuspended with 10 ml PBS and further centrifuged as mentioned above, the last step repeated twice. Transcription from RNA into cDNA was performed after transfer of the pellet into an Eppendorf cup and the RT protocol was used as described for tracheal transcription (see above).

### RNA extraction

The excised tracheae were homogenized in TRIzol (Life Technologies, Carlsbad, USA) using a bead mill and subsequent RNA extraction was performed using a combination of guanidinium thiocyanate-phenol-chloroform separation followed by column-based RNeasy Plus Mini Kit (Qiagen, Hilden, Germany) extraction according to the manufacturer’s instructions. Briefly, each trachea was individually homogenized in 1 ml of TRIzol, then 200 μl chloroform were added, mixed and after 3 minutes incubation at room temperature centrifuged at 12.000 *x g* for 15 minutes at 4°C. The aqueous phase containing the RNA was removed and directly used for the further extraction using the binding column. RNA was eluted from the column with 30 μl RNAse-free water and stored at -80°C until further analysis.

### Quantitative PCR

RNA concentration was photometrically measured and 350 ng of each preparation was reverse transcribed using the QuantiTect Reverse Transcription Kit (Qiagen, Hilden, Germany) according to the manufacturer’s instructions including a genomic DNA removal step and a prolonged RT synthesis step to ensure proper transcription of difficult RNA species. Subsequent Real time PCR analysis was performed on a StepOnePlus™ PCR cycler (Applied Biosystems, Weiterstadt, Germany). Following pre-designed TaqMan assays were used: mouse 18S (assay ID Mm03928990_g1) and mouse TNFR1a (assay ID Mm00441883_g1). PCR reactions were set up using the TaqMan Universal PCR Master Mix (Applied Biosystems, Weiterstadt, Germany). All experiments were run in triplicate. Results are interpreted by calculating the 2^ΔCt^ (ΔCt: Ct 18S – Ct TNFR1a) of each sample.

### Immunohistochemistry

For immunohistochemistry mice were sacrificed by an overdose of isoflurane, after confirmation of death the tracheae were harvested as described in previous sections. The organs were transferred into 4% paraformaldehyde (PFA) in phosphate buffer solution pH 7.2 for immersion fixation and stored overnight at room temperature (21°C). After rinsing in phosphate buffer the tissue was cryoprotected using 18% sucrose and then snap frozen in Tissue Tek. Cryosections 10 μm were cut and mounted on glass slides, air dried and subsequently incubated for 1 h using 10% normal horse serum containing 0.5% Tween 20, 0.1% BSA in PBS, pH 7.4. After removal of the blocking solution the sections were incubated with the primary antibody against TNF-R1 (1:400 rabbit polyclonal, abcam, Cambridge, UK) overnight at room temperature. Afterwards the antibody solution was rinsed off using phosphate buffer. Secondary antibody was applied for 1 h at room temperature using donkey anti rabbit 1∶2000 linked with Cy3 (Chemicon, Hampshire, UK). Afterwards the secondary antibody was rinsed off in phosphate buffer and then the sections were further fixed in 4% PFA in 0.1 M phosphate buffer for 10 min. and rinsed again in phosphate buffer and finally coverslipped in carbonate buffered glycerol (pH 8.6). The tracheal sections were evaluated using an epifluorescence microscope using a 40x lens (Axioplan 2 imaging; Zeiss, Jena, Germany) fitted with the appropriate filter sets. Specificity of the primary antibody against TNF-R1 (1:400) was tested by re-absorption with the TNF-R1 peptide (60 μg/100 μl, Thermoscientific, Schwerte, Germany) at 8°C for 24 hrs. After this incubation the sample was centrifuged for 1 min at 1000 rpm and then the tracheal sections were incubated at room temperature over night. All other incubation steps were performed identical as described for immunohistochemical staining.

### Statistical analysis

Certain pre-defined time points of different experiments were compared using Mann-Whithney test. Statistical significance was assumed when p<0.05.

## Results

### TNF- α increases doses dependent cilia driven PTV

All experiments were conducted using the same experimental design: A resting period for 30 minutes (not shown) was used to achieve a steady-state condition before stimulation, which started 60 minutes after confirmed death. Measurements were performed over a period of 60 minutes. Vitality was proofed at the end of each experiment by application of ATP (10^−5^ M) at minute 60 and further data sampling over 10 minutes (figure 1C).

**Figure 2:**
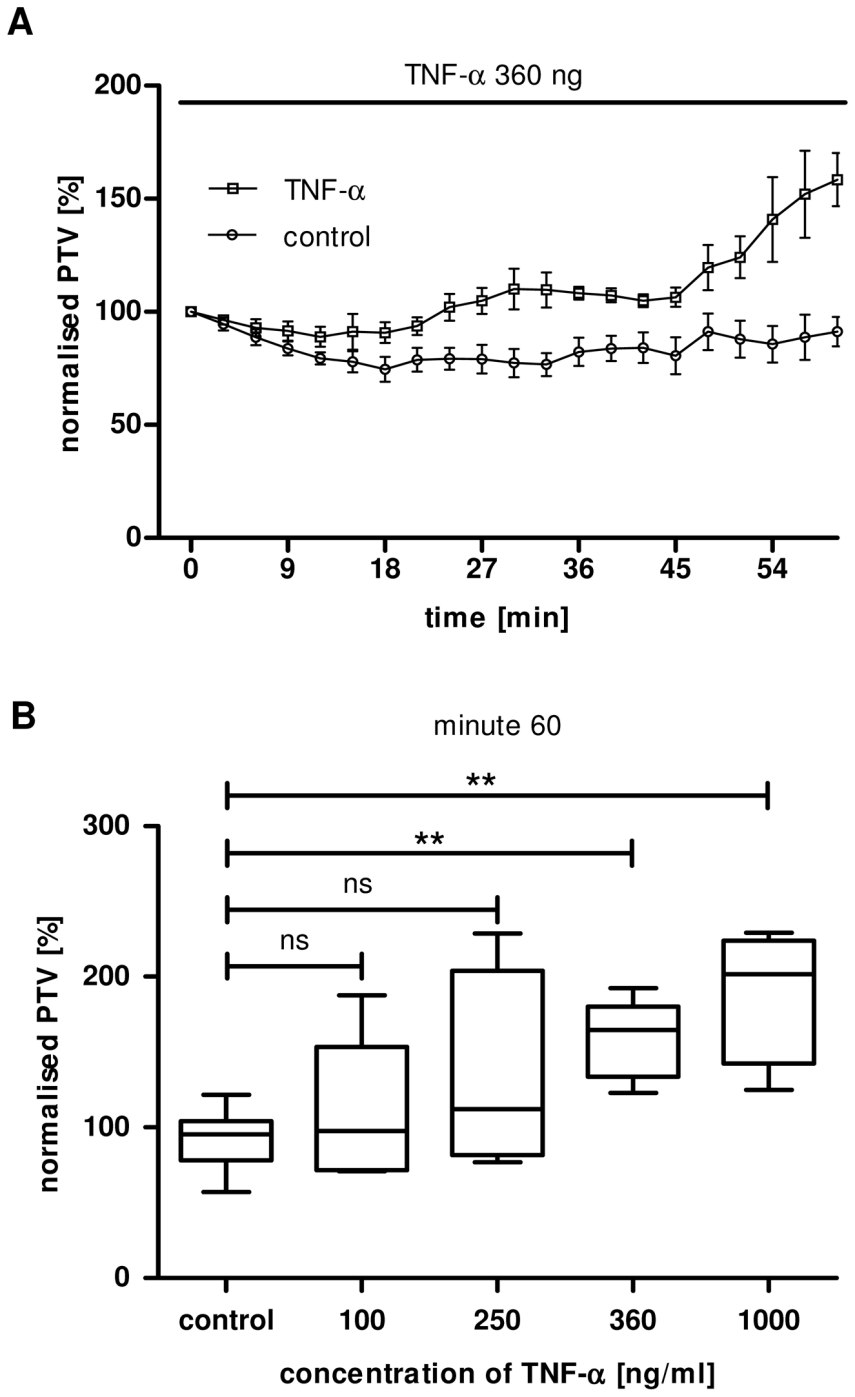
**A:** TNF-α induced increase in PTV**.** Exposure to TNF-α (360 ng/ml) induced an increase in PTV with a delay of approximately 24 min, the increase in PTV was already significant after 30 min of application (p<0.05). After a brief plateau in which the increased PTV did not reach the threshold of statistical significance, it further rose, still in the presence of TNF-α, from minute 51. It eventually reached its maximum between 51 and 60 minutes after TNF-α application (p<0.001, vitality test not shown, TNF-α: 360 ng/ml, n = 5, Mean ± S.E.M.). **B:** Concentration dependency of TNF-α induced PTV. Cilia driven PTV is shown during the application of TNF-α at minute 0. Application of TNF-α 100 ng/ml (n = 9) or 250 ng/ml (n = 4) showed a trend to enhance cilia driven PTV compared to control (n = 9; p = 0.4). The PTV increased significantly until minute 60 during continuous stimulation with 360 ng/ml TNF-α (n = 5, p<0.01, compared to control) or 1000 ng/ml (n = 4, p<0.01, compared to control, Mean ± S.E.M.) Kolmogorow-Smirnow-test followed by Mann Whitney U-test.

Tracheae were stimulated 60 minutes after isoflurane induced death with TNF-α in different concentrations (100 ng/ml, 250 ng/ml, 360 ng/ml and 1000 ng/ml). PTV was arbitrarily set as 100% for minute 0 to normalise for inter-individual variability (figure 2A).

Application of 100 ng/ml (p = 0.59) or 250 ng/ml (p = 0.39) TNF-α did not result in an increase of the PTV compared to control. In contrast, higher concentrations with 360 ng/ml (p<0.01) or 1000 ng/ml (p<0.01) TNF-α evoked a significant increase in cilia driven particle transport compared 60 minutes after TNF-α application with the control group (figure 2B).

### TNF-α enhanced CBF increases PTV

**Figure 3:**
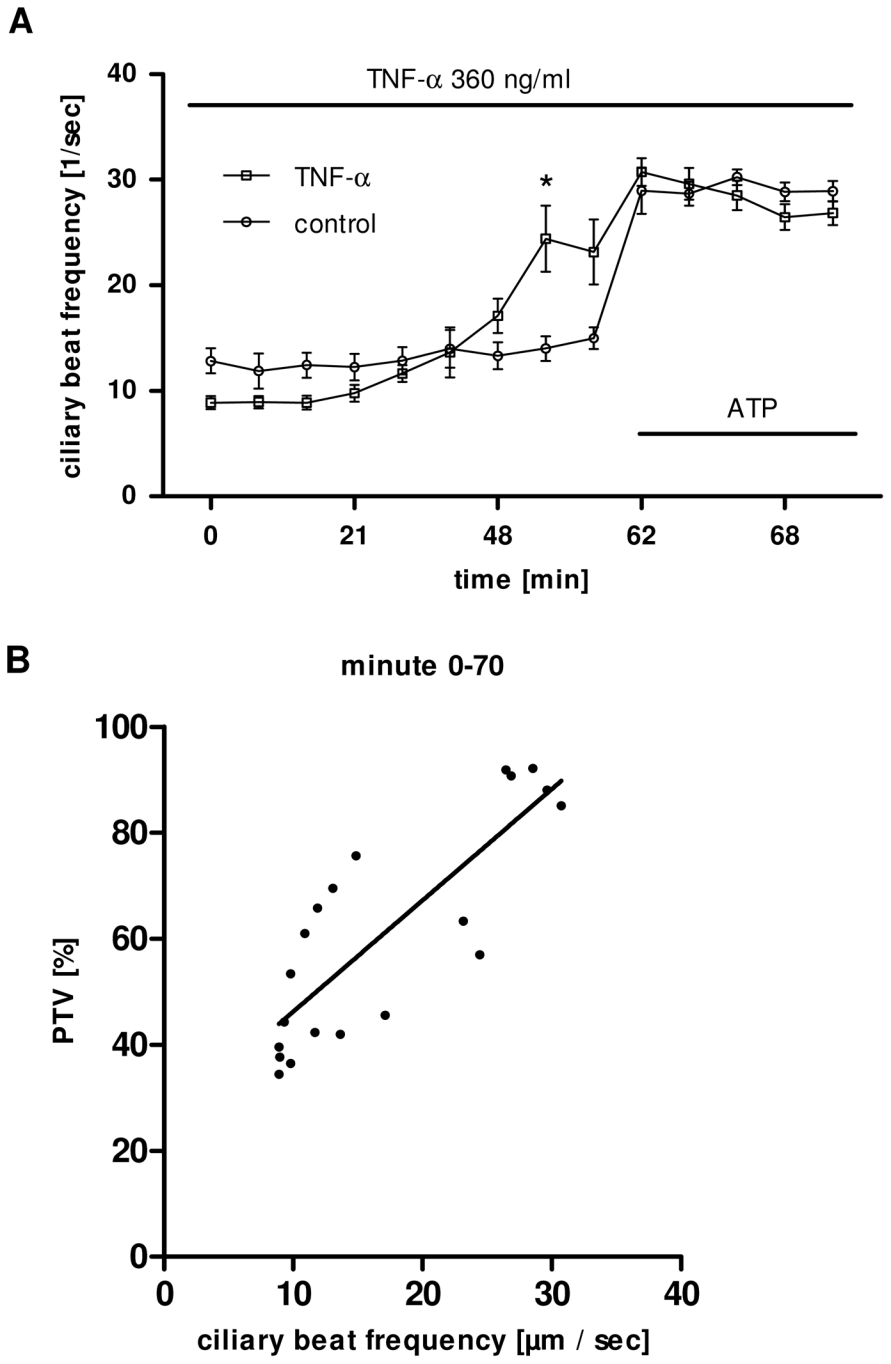
**A:** TNF-α increases CBF. Under control conditions no changes in CBF were observed. Here only the application of TNF-α (360 ng/ml, n = 5) at minute 0 is shown this responded by a rapid increase in CBF, that became significant from minute 48 (p<0.05), reaching its maximum at minute 57 (before ATP application, p<0.05) compared to control (n = 5). (Mann Whitney U-test (Mean ± S.E.M.). **B:** Correlation of cilia driven particle transport with CBF. Increase of CBF (mean values; n = 5) during the exposure to TNF-α (360 ng/ml) strongly correlates (r = 0.83, p<0.001) with the parallel increase in PTV (mean values; n = 5) during the chosen observation period (Spearman correlation test for nonparametric correlation).

Under control conditions CBF remained stable throughout the entire experimental period. Application of TNF-α (360 ng/ml) led to an increase of the CBF that became significant from minute 48 until minute 60 (p<0.01, figure 3A).

TNF-α-induced increase of CBF strongly correlates (r = 0.83, p<0.001) with the parallel increase in PTV during the stimulation period and the vitality phase (figure 3B). This suggests that in these experiments an enhanced CBF is the underlying mechanism of higher transport velocities.

### Evidence for tracheal TNF-R1 mRNA expression

**Figure 4:**
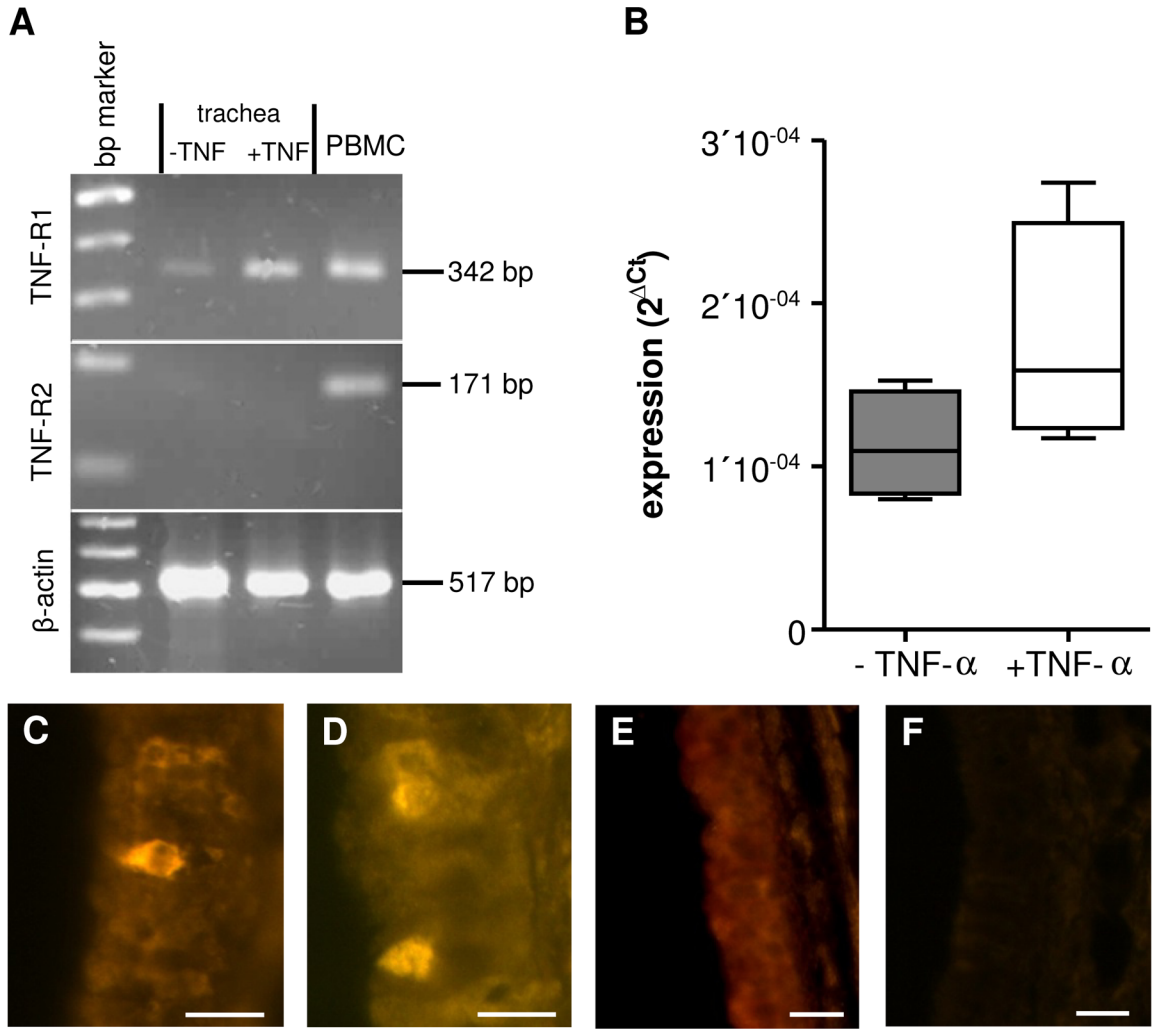
Expression of TNF-R1 in tracheal epithelium. **A:** In RT-PCR experiments using whole tracheal preparations we found evidence for mRNA of TNF-R1 but not TNF-R2 (n = 3, bp: 100 kD). **B:** In tracheae previously exposed to TNF-α over 60 min we observed a non-significant increase in TNF-R1 mRNA when using qPCR (p = 0.2, n = 4). **C:** In addition immunoreaction against TNF-R1 was used to identify the anatomical location of the TNF-R1. Immunoreaction against TNF-R1 stained only cells within the epithelial layer. These were either found as isolated cells or as clusters of 2–4 cells (**D**). **E:** Pre-absorption of the primary antibody with TNF-R1 peptide did not stain any cells in the tracheal epithelium. **F:** In addition experiments omitting primary antibody were used as negative controls evoked no immunoreactive staining of epithelial structures. (Scale bars: 20 μm).

To further evaluate which receptors are involved in the TNF-α induced mechanism we performed both RT-PCR and qPCR experiments on freshly isolated tracheae and on organs that had previously been stimulated with TNF-α during CBF or PTV experiments. In control experiments using freshly harvested whole tracheae we detected mRNA of the TNF-R1 but not of TNF-R2. TNF-R2 mRNA was neither detected in control nor in TNF-α treated tracheae (RT-PCR; n = 3; figure 4A). In TRIzol homogenated tracheae (again using whole tracheal walls) that had been exposed to TNF-α (360 ng) over 60 min the gene expression of TNF-R1 was detectable. In these tracheae the TNF-R1 mRNA showed a trend to be increased using qPCR. However, this rise in mRNA reached not statistical significance (p = 0.2, n = 4, figure 4B).

### The TNF-R1 is exclusively located in tracheal epithelium

In order to investigate the location of cells expressing the TNF-R1 peptide in the entire trachea, immunohistochemical stainings of tracheae were performed. In fact, incubation with an antibody against TNF-R1 led to a high enrichment of immunoreactivity in small cells embedded in the epithelium (figure 4C). These TNF-R1 immunoreactive positive cells were exclusively located in the tracheal epithelium and were either isolated or occurred in small groups (2–4 cells), (figure 4C–D). The specificity of the antibodies was proofed using both pre-absorption with TNF-R1 peptide and omitting of the primary antibody (figure 4E, F).

### TNF-α-induced changes are TNF-R-1-dependent

**Figure 5:**
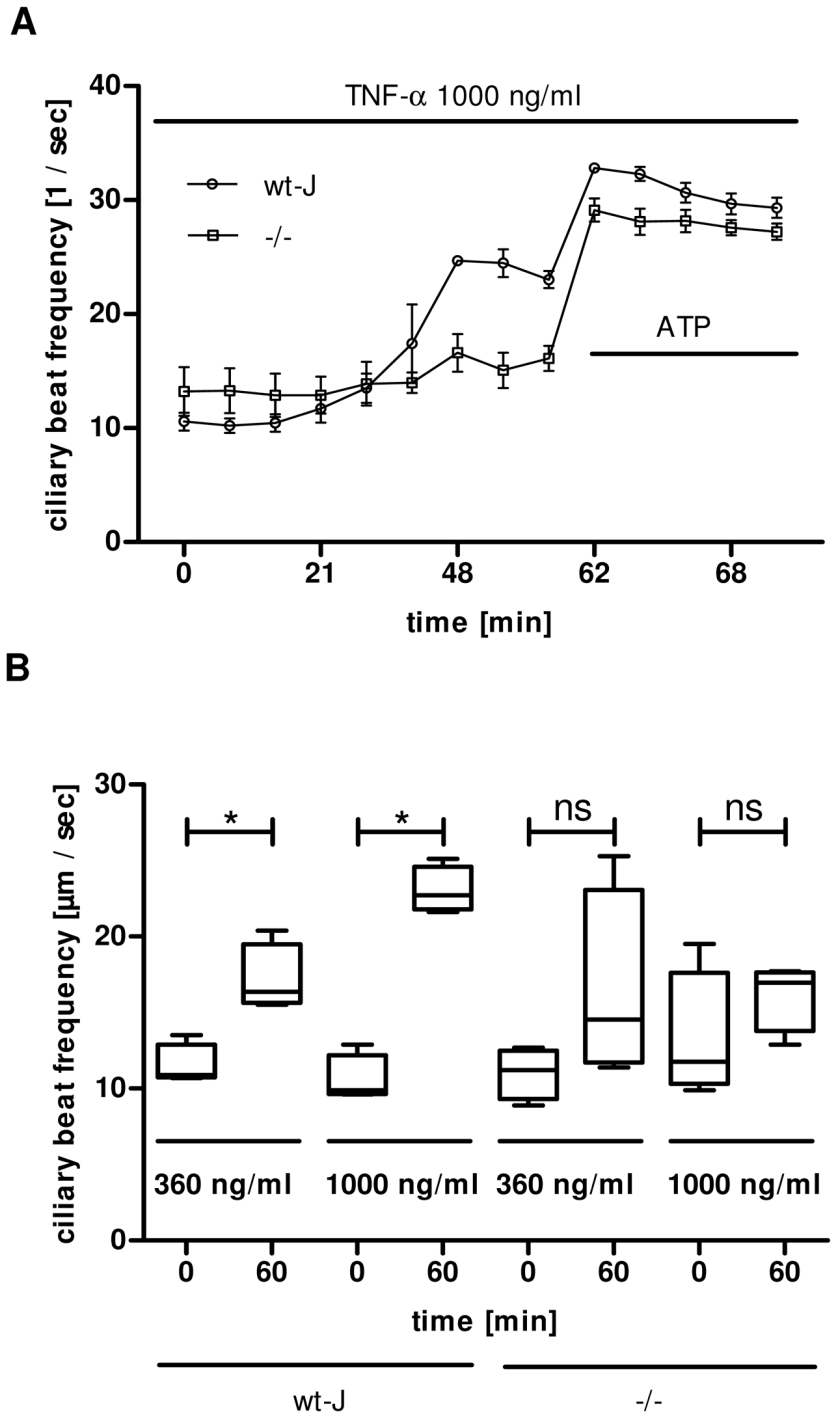
TNF-α effects are reduced in TNF-R1^−/−^ mice. **A:** The recording shows the highest concentration of TNF-α (1000 ng/ml) applied. In wt-J mice it resulted in an increase of the CBF becoming significant between minute 48 and minute 60 compared to TNF-R1^−/−^ mice using Mann Whitney U-test (p<0.05, Mean ± S.E.M.). **B:** The dose dependency of TNF-α induced increase in CBF in wt-J mice became significant between minute 60 and minute 120 (p<0.05). This significant difference can not be provoked, when TNF-α is applied at TNF-R1^−/−^ tracheae (p>0.05, Mann Whitney U-test. Mean ± S.E.M., n = 40 cells from 4 tracheae out of 4 animals).

TNF-α 1000 ng/ml led to a significant CBF elevation in wild-type (wt-J) mice. In contrast, the same concentration TNF-α could not provoke an increase in CBF in TNF-R1 knock-out mice (–/–) (figure 5A). The concentration dependent reaction of TNF-α of 360 ng/ml or 1000 ng/ml showed a significant difference between minute 0 and minute 60 in wt-mice. When TNF-α was applied at minute 0 neither 360 ng/ml nor 1000 ng/ml caused a significant enhancement at minute 60 (figure 5B).

### Methysergide and cyproheptadine block the TNF-α induced increase in PTV

**Figure 6:**
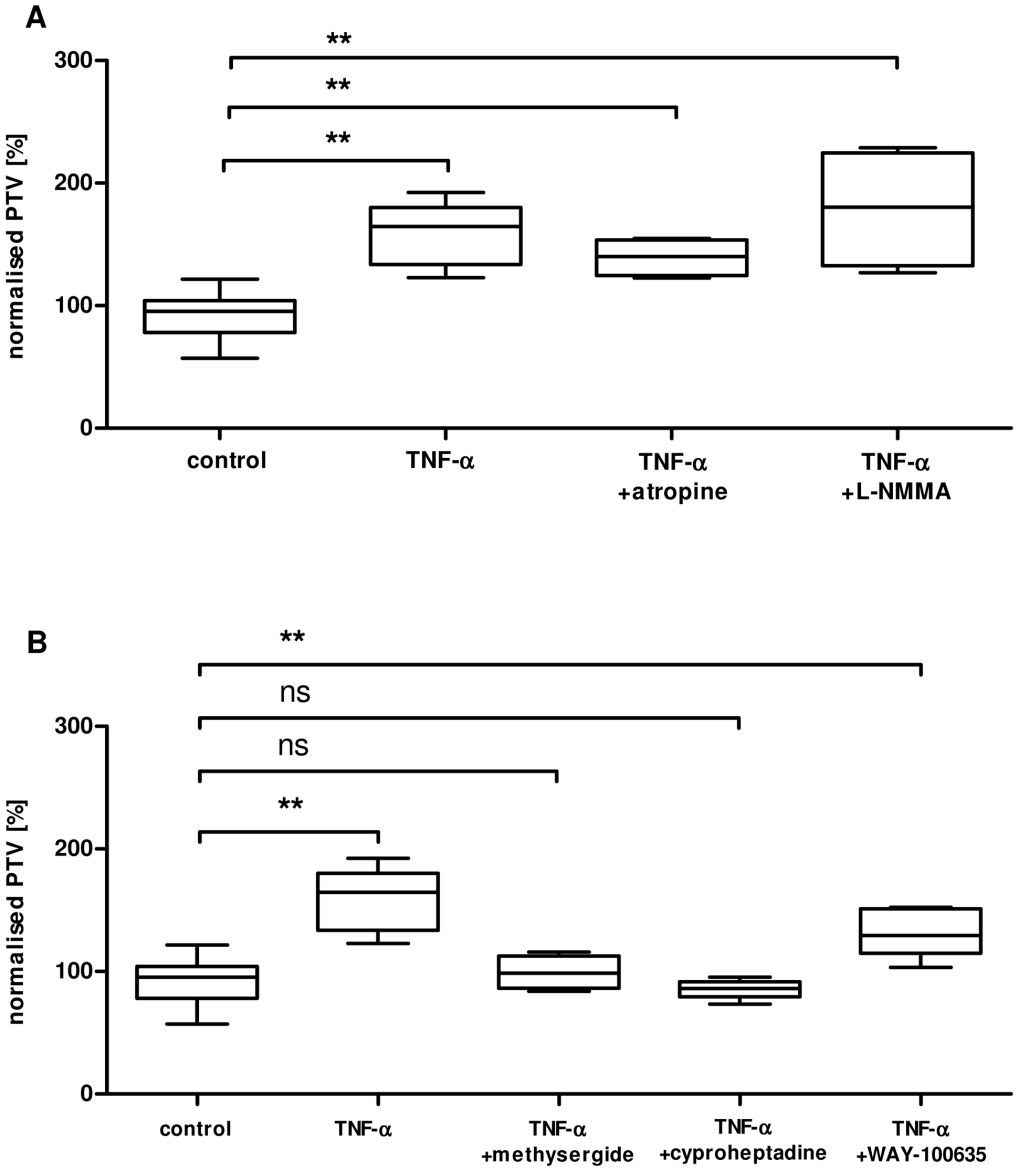
Inhibition of TNF-α induced PTV by serotonin receptor blockade. Mice tracheae were analysed at minute 60. **A:** Atropine (1 μM, n = 4) or L-NMMA (100 μM, n = 4) did not prevent the increase in PTV as a response to TNF-α (360 ng/ml) application. **B:** The serotonin antagonist methysergide (100 μM) reduced the TNF-α (360 ng/ml) evoked increase in PTV (p<0.05). In contrast 1 μM WAY-100635 (a specific 5-HT_1A_ receptor antagonist) did not reduce the TNF-α evoked increase in PTV (p = 0.22). Cyproheptadine (specific 5-HT_2_ receptor antagonist, 5 μg/ml) blocked the TNF-α induced PTV elevation (p<0.01, n = 9 in control, n = 5 in all other experiments, Mann Whitney U-test. Mean ± S.E.M.).

The PTV of the tracheae were compared 60 minutes after the stimulation with TNF-α (360 ng/ml) and a pre-incubations with either a NOS inhibitor (L-NMMA) or a muscarinic receptor blocker (atropine) were performed. Neither atropine (1 μM), nor L-NMMA (100 μM) or L-NAME (100 μM data not shown) could prevent a TNF-α induced increase in the PTV of tracheal epithelium (figure 6A). Administration of atropine (1 μM, n = 4, p = 0.31) or L-NMMA (100 μM, n = 4, p = 0.19) omitting TNF-α had no effect on PVT when compared to control (data not shown). In addition a serotonin related effect was analysed using different pharmacological agents. The unspecific inhibitor methysergide (100 μM) completely reduced the TNF-α evoked increase in PTV (p<0.05, n = 4). Furthermore we administered specific serotonin receptor antagonists. WAY-100635 (1 μM, specific inhibitor of the serotonin receptor 5-HT_1A_) did not alter the response to TNF-α (p = 0.22, n = 5), wereas cyproheptadine (14 μM, specific inhibitor of 5-HT_2_) completely inhibited the TNF-α evoked increase in PTV (p<0.01, n = 5, figure 6B). Compared to control experiments neither methysergide (p = 0.85), cyproheptadine (p = 0.4) nor WAY-100635 (p = 0.4) had a significant influence on PTV when applied without TNF-α.

## Discussion

In the present study we provided evidence for an early TNF-α triggered increase in PTV and CBF. The mucociliary clearing system is an important innate lung protective mechanism to disburden the airway surface area from debris and pathogen particles. In addition to the active mechanical clearing, a specific composition of mucus covers as a thin liquid layer the entire epithelium of lower airways and lungs to reduce the surface tension. Additional the surfactant contains ATP, that has been released by pulmonary epithelial cells, which stimulates purinergic receptors P2Y on apical membranes of ciliary bearing epithelial cells [13] [2] [14] [15]. In these epithelial cells the soluble adenylyl cyclase is activated in close vicinity to the cilia, it synthesizes cAMP subsequently enhancing CBF [16]. This oral directed ciliary beating as well as the fine regulated composition of the airway liquid layer together play an important well coordinated role in the clearing mechanism of lower airways.

Nevertheless, additional different physiological factors like the neuromediators acetylcholine, adrenaline and serotonin are known as constant regulators to maintain the permanent ciliary beating. Beside these classical modulators, under certain circumstances further physiological or patho-physiological conditions can alter ciliary function [1] [17] [8]. During inflammation cytokines play a key role in regulation of body function and among these cytokines TNF-α contributes to multiple distinct pathways. Beyond its pyrogenic effect as an inflammatory mediator [18], TNF-α also functions as a major immunological trigger. It has a broad influence in many different inflammatory diseases [19] [20]. In a previous study it has been shown that TNF-α induces under long term exposure a NO mediated increase in ciliary motility of isolated and cultivated bovine airway epithelium cells [21]. In the present study we observed that TNF-α already triggered during short term exposure an elevation of both CBF and PTV. This elevation of CBF and PTV of the whole intact tracheal epithelium was independent from NO depending mechanisms that have been described under long term exposure (see below).

Correspondent we observed a dose dependent effect of TNF-α on ciliary bearing cells (figure 2B), that became obvious under constant exposure to TNF-α within 60 minutes. A dose and time related cell response to TNF-α application is heterogeneous, and depends on the chosen cell lines, but seems to be a common effect as it has been previously described. In the rhabdomyosarcoma cell line KYM-1 exposure to TNF-α induces dose dependent apoptosis via a pro-apoptotic caspase activation and in parallel it triggers the nuclear factor κB (NF-κB)-mediated survival pathways [22]. Furthermore TNF-α-effects onto immune cells seem also dose dependent, high doses evoked maximal adhesion of murine progenitor T cells to murine arterial endothelial cells, to human umbilical endothelial cells or to bovine aortic endothelial cells [23]. TNF-α may not only activate genuine immune cells, thus it also activates cell types closely involved in inflammatory defence mechanisms. The results of the present study not only provided evidence for increased CBF. Moreover we measured increased mechanical transport capacity in different experimental setups strongly correlating to the change in CBF (see figure 3), which corresponds to earlier findings of an elevation of CBF of isolated bovine cells exposed to TNF-α after 24 hrs [21]. However, here we used organ explant experiments to achieve in vivo related conditions, in which interaction and effective TNF-α concentrations may differ from isolated ciliary bearing cells. According to the close location between TNF-α producing cells, like alveolar macrophages or other pro-inflammatory cells, and their target cells, we applied higher TNF-α concentrations to achieve nearly realistic conditions. In bronchoalveolar fluid of human patients with acute respiratory distress syndrome TNF-α concentrations can reach ranges above 10 ng/ml [24], but even higher local concentrations ranging from 0.6 to 2400 ng/ml have been found between intact cell structures [25]. For that reason we analysed the effects of TNF-α concentrations in the range from 0 to 1000 ng/ml, which turned out to be approved TNF-α concentrations similar to local cell constellations including TNF-α producing cells. [25], [26].

Currently two TNF-α receptors are known that transpond the effects to its target cells. The main target of TNF-α seems to be the plasma membrane-bound receptor TNF-R1, which is expressed in various cell types. However, the TNF-R2 is assumed to be located only on immune cells, but may also alter organ function, via these activated cells.

The mRNA of TNF-R1 was detected in whole trachea using RT-PCR or qPCR. Immunohistochemical staining against TNF-R1 protein revealed the location of this receptor being exclusively located within the epithelium of the trachea under non-stimulated control conditions (figure 4). In qPCR experiments we observed an upregulation of TNF-R1 mRNA during exposure to TNF-α, albeit it reached not statistical significance during the chosen exposure period. Most probably the experimental setup of 60 min. exposure time was too short to trigger a significant upregulation of the TNF-R1 mRNA when using qPCR. Hence, we obtained no evidence for the expression of TNF-R2 neither in control nor in TNF-α treated tracheae (figure 4). Since, TNF-R1 mRNA was detected when using whole trachea, we applied immunohistochemical staining to localize the TNF-R1 protein. Our experiments clearly provided evidence that the TNF-R1 protein is located within few cells of the tracheal epithelium. This staining was proofed by an adequate control for the specificity of the primary TNF-R1 antibody using pre-absorption with the TNF-R1 peptide and omission of the primary antibody in a further series of experiment. According to these present results, we assume that TNF-R1 is exclusively expressed in the tracheal epithelium.

In experiments using TNF-R1^−/−^ mice the response to TNF-α was almost completely diminished, confirming a signal transduction pathway via TNF-R1. Additionally the concentration dependent effect to TNF-α application (360 ng/ml; 1000 ng/ml) was extinguished in TNF-R1^−/−^ mice. Activation of TNF-R1 seems to be the main pathway of TNF-α evoked responses in cilia bearing cells which are non immunological cells. Further TNF-R1 plays not only in the tracheal epithelium a pivotal role for cell activation but also in lower airway smooth muscle cells. Despite the coexistence of both TNF-R2 and TNF-R1 in this cell line, the latter plays the key role for activation of nuclear factor-κB transcription factor function or calcium-signal potentiation and contributes to overreaction of bronchial airway muscle during inflammation [27] [28] [29]. These observations are in line with our findings, indicating that TNF-R1 is the principle signal converter for TNF-α induced activation of ciliary function.

Ciliary function is conducted continuously by physiological neuromediators released from autonomic nerve fibres or from adjacent cells in a paracrine pattern [8]. Alternative modulators deriving from sources different from the above mentioned may also contribute to the regulation of airway cell functions. Stimulation of mice tracheal epithelial cells e. g. with serotonin triggers a release of acetylcholine from epithelial cells resulting in a subsequent contraction of the underlying smooth muscle cell layer [7]. However on the one hand application of acetylcholine increases rapidly CBF [30], on the other hand serotonin also mediates an increase in CBF independent from the release of acetylcholine [31]. Since both acetylcholine and serotonin seem to generate independently an increase in ciliary activity we observed the independent effects of both on cilia function. Among a cholinergic stimulation of cGMP, which constitutes equal to cAMP, an important messenger to activate a phosphorylation and mediate an increase in cilliary beat frequency, NO induces the synthesis of cGMP based on activation of soluble guanylyl cyclase [32] [33] [34] [4]. But neither inhibition of NO generation in the present study using L-NMMA or L-NAME did prevent the TNF-α induced increase in PTV, nor a muscarinic receptor blockade using atropine was able to reduce or inhibit the response to TNF-α. Yet application of methysergide an unspecific serotonin antagonist completely prevented the TNF-α induced increase in CBF and PTV. In addition only cyproheptadine a selective 5-HT_2_ serotonin receptor antagonist but not WAY-100635 a selective 5-HT_1A_ serotonin receptor antagonist could inhibit the TNF-α induced ciliary activation (figure 6). This strongly indicates that serotonin may contribute in this activation cascade via its receptor 5-HT_2_.

Serotonin is a derivative from tryptophan and known as a neurotransmitter released mainly from neuroendocrine cells. It is as well a regulator for various biological processes even outside the central nervous system. It has direct influence on the cardiovascular system, e.g. regulation of the heart rate. Especially in the gastrointestinal tract serotonin plays an important role, here it stimulates intestinal peristalsis and intestinal secretion. In the lung serotonin is involved in the pathogenesis of pulmonary hypertension, during hypoxia the intravascular serotonin concentration increases which then results in a rise in 5-HT_2B_ receptor signaling of endothelial cells from pulmonary arteries consequently increasing vascular resistance [35] [36] [37]. In addition serotonin as well induces pulmonary artery remodeling by hypertrophy of smooth muscle cells, this goes in parallel to the enhanced expression of the serotonin transporter (SERT) [38] [39] [40]. This mechanism is similar to the activation of platelet aggregation by SERT [41] [42]. Both link inflammatory processes with different cell functions that are primarily not associated to the specific immune system, similar to our observation. So far we assume that TNF-α induces the increase of PTV and CBF by serotonin release, since we were able to completely inhibit the response to TNF-α by pharmacological inhibition of serotonin receptors in general and specifically by the inhibition of 5-HT2. We further presume that serotonin is released from granules probably deriving from mast cells that are located in the trachea, thus this has to be clarified in further studies [31]. In addition to the fact that mast cells release TNF-α under inflammatory conditions, they themselves can be stimulated by TNF-α via binding to TNF-R1 [43]. Likewise, serotonin derived from mast cells in the mouse trachea are known to activate 5-HT_2_ receptors on parasympathetic cholinergic neurons, which lead to acetylcholine release and airway smooth muscle contraction [44]. In our experiments the TNF-α induced increase in PTV and CBF was completely inhibited by methysergide, an unspecific serotonin receptor blocker and cyproheptadine a specific 5-HT_2_ serotonin receptor blocker. Therefore we assume an early TNF-α induced serotonin dependent action onto the ciliary activity, that is independent from the late increase in CBF as has been reported for isolated and cultivated bovine cilia bearing cells [21]. Furthermore in our experimental set up inhibition of NO or acetylcholine dependent signaling pathways did not interact with the observed TNF-α-stimulation. Thus, we assume that serotonin derives from mast cells and triggers the increases in PTV and CBF via the 5-HT_2_ serotonin receptor.

In conclusion we assume that TNF-α induces an increase in PTV and CBF by binding to TNF-R1 on tracheal mast cells which then release serotonin that stimulates the 5-HT_2_ serotonin receptor on cilia bearing cells. This cascade may function as a protective mechanism during early periods of inflammation or developing pneumonia.
